# The N-terminus of the *Chlamydia trachomatis* effector Tarp engages the host Hippo pathway

**DOI:** 10.1128/spectrum.02596-24

**Published:** 2025-03-10

**Authors:** George F. Aranjuez, Om Patel, Dev Patel, Travis J. Jewett

**Affiliations:** 1 Immunity and Pathogenesis Division, Burnett School of Biomedical Sciences, College of Medicine, University of Central Florida 124507 , Orlando, Florida, USA; University of Nebraska Medical Center, Omaha, Nebraska, USA

**Keywords:** *Chlamydia trachomatis*, effector functions, host-pathogen interactions, *Drosophila*, cell signaling

## Abstract

*Chlamydia trachomatis* (*Ct*) is an obligate, intracellular Gram-negative bacteria and the leading bacterial sexually transmitted infection in the United States. *Chlamydia* manipulates the host cell biology using various secreted bacterial effectors during its intracellular development. The early effector *t*ranslocated *a*ctin-*r*ecruiting *p*hosphoprotein (Tarp), important for *Chlamydia* entry, has a well-characterized C-terminal region which can polymerize and bundle F-actin. In contrast, not much is known about the function of the N-terminus of Tarp (N-Tarp), though present in many *Chlamydia* spp. To address this, we use *Drosophila melanogaster* as an *in vivo* cell biology platform to study N-Tarp-host interactions. Transgenic expression of N-Tarp in *Drosophila* results in developmental phenotypes consistent with altered host Salvador-Warts-Hippo signaling, a conserved signaling cascade that regulates host cell proliferation and survival. We studied the N-Tarp function in larval imaginal wing discs, which are sensitive to perturbations in Hippo signaling. N-Tarp causes wing disc overgrowth and a concomitant increase in adult wing size, phenocopying overexpression of the Hippo co-activator Yorkie. N-Tarp also causes upregulation of Hippo target genes. Last, N-Tarp-induced phenotypes can be rescued by reducing the levels of Yorkie or the Hippo target genes *CycE* and *Drosophila inhibitor of apoptosis 1 (Diap1)*. Thus, we provide evidence that the N-terminal region of the *Chlamydia* effector Tarp is sufficient to alter host Hippo signaling and acts upstream of the co-activator Yorkie.

**IMPORTANCE:**

The survival of obligate intracellular bacteria like *Chlamydia* depends on the survival of the host cell itself. It is not surprising that *Chlamydia*-infected cells are resistant to cell death, though the exact molecular mechanism is largely unknown. Here, we establish that the N-terminal region of the well-known *Ct* early effector Tarp can alter Hippo signaling in vivo. Only recently implicated in *Chlamydia* infection, the Hippo pathway is known to promote cell survival. Our findings illuminate one possible mechanism for *Chlamydia* to promote host cell survival during infection. We further demonstrate the utility of *Drosophila melanogaster* as a tool in the study of effector function.

## INTRODUCTION

*Chlamydia trachomatis* (*Ct*) is a Gram-negative, obligate intracellular bacteria and is the leading cause of infection-induced blindness globally and the most commonly reported sexually transmitted infection (STI) in the United States, far ahead of other STIs such as gonorrhea and syphilis. Moreover, the year-over-year incidence of *Chlamydia* infections is consistently increasing (1), representing an ever-increasing public health burden. Despite its prevalence, much of the molecular strategies that *Chlamydia* employs to promote infection are not completely understood.

Host cell invasion is a crucial first step in *Chlamydia* infection. After attaching to the surface of the host cell, *Chlamydia* injects several bacterial effectors via type III secretion system needles (2, 3). These “early effectors” have unique but synergizing functions that promote efficient host cell invasion. The most well-characterized early effector is *t*ranslocated *a*ctin-*r*ecruiting *p*hosphoprotein (Tarp). It has been shown, through various approaches, that Tarp is required for efficient host cell invasion (4–6). The recruitment and remodeling of host actin is a common mechanism employed by intracellular bacteria such as *Chlamydia* to gain entry into the host cell (7–9). *In vitro*, Tarp has been shown to promote the formation of F-actin as well as bundle existing F-actin filaments via different functional domains on the C-terminus (10, 11). Other early effectors such as TmeA complement Tarp function by promoting actin filament formation via the host Arp2/3 complex (12, 13), while the early effector TmeB acts to fine-tune TmeA activity (14).

Tarp is a ~1,000 amino acid protein with distinct N- and C-termini. Tarp’s actin-remodeling domains are found on its C-terminus, while the N-terminus is largely devoid of annotated functional domains. Interestingly, this seemingly unassuming N-terminal configuration is observed in Tarp protein from multiple *Chlamydia* spp. that target a variety of host organisms (15). For *C. trachomatis*, the N-terminus of Tarp (N-Tarp) contains tyrosine-rich repeats that are rapidly phosphorylated upon delivery into the host cell (8, 16, 17). The functional contribution of N-Tarp during *Chlamydia* infection is still not clearly understood, though tyrosine phosphorylation is a potent post-translational modification that can engage a wide variety of signaling pathways.

We recently developed a new way to investigate effector function *in vivo* using the model organism *Drosophila melanogaster* as a cell biology platform. Via transgenic expression of *Chlamydia* effectors in flies, we are able to study effector function in isolation, away from the confounding effects of active infection. Furthermore, genetic tools in *Drosophila* research allow for targeted expression of effectors in the cell or tissue of interest. Last, taking advantage of the well-understood developmental biology of *Drosophila*, we can infer effector function from the phenotypes caused by effector expression in fly tissues. We have successfully used this platform to verify that Tarp’s F-actin bundling activity also occurs *in vivo* and that Tarp can displace endogenous F-actin bundlers (18).

We then employed this platform to uncover N-Tarp function *in vivo* in an unbiased approach. We observed that transgenic expression of N-Tarp in flies displayed phenotypes (bristle duplication) consistent with disruption of the Hippo pathway, serving as a launching point to investigate the state of Hippo signaling in *Ct*-infected cells *in vitro* (19, 20). Indeed, changes in key members of the Hippo core components, as well as gene expression of canonical Hippo target genes, were observed during *Ct* infection in a Tarp-dependent manner (19).

First described in *Drosophila*, the Hippo pathway is a highly conserved signaling pathway that controls cell proliferation and cell survival, ensuring proper organ size control during development (21). The core signaling pathway is a kinase cascade that controls the nuclear localization of the transcription co-activator Yorkie (YAP/TAZ in mammals), which, together with the DNA-binding protein Scalloped (TEAD in mammals), acts as a transcription factor to drive downstream target genes such as *Cyclin E* and *Drosophila inhibitor of apoptosis 1* (*Diap1*) (*XIAP* in mammals) that regulate the cell cycle and apoptosis, respectively (see diagram in Fig. 5). Originally discovered and characterized in *Drosophila*, perturbations in Hippo signaling lead to overgrowth or undergrowth of organs or even whole organisms (22). Outside of its role in animal development, the Hippo pathway is also involved in wound healing and underlies several types of cancer (23, 24). In the context of *Chlamydia* infection, we indeed found evidence of changes in the Hippo pathway during infection and that these changes occur in a Tarp-dependent manner (19).

In this study, we extend our initial observation in Shehat et al. (19) by using *Drosophila* wing development as a model to study the interplay between N-Tarp and the Hippo pathway *in vivo*. The N-Tarp expression in the developing wing leads to an increase in size, consistent with altered Hippo signaling. We use anatomical, molecular, and genetic approaches to establish that N-Tarp acts through the co-activator Yorkie to upregulate Hippo target genes. This work attributes a new function to the poorly understood N-terminal region of the early effector Tarp as a means for *Chlamydia* to manipulate the host Hippo signaling.

## RESULTS

### Expressing N-Tarp in the wing imaginal disc results in tissue overgrowth

**Fig 1:**
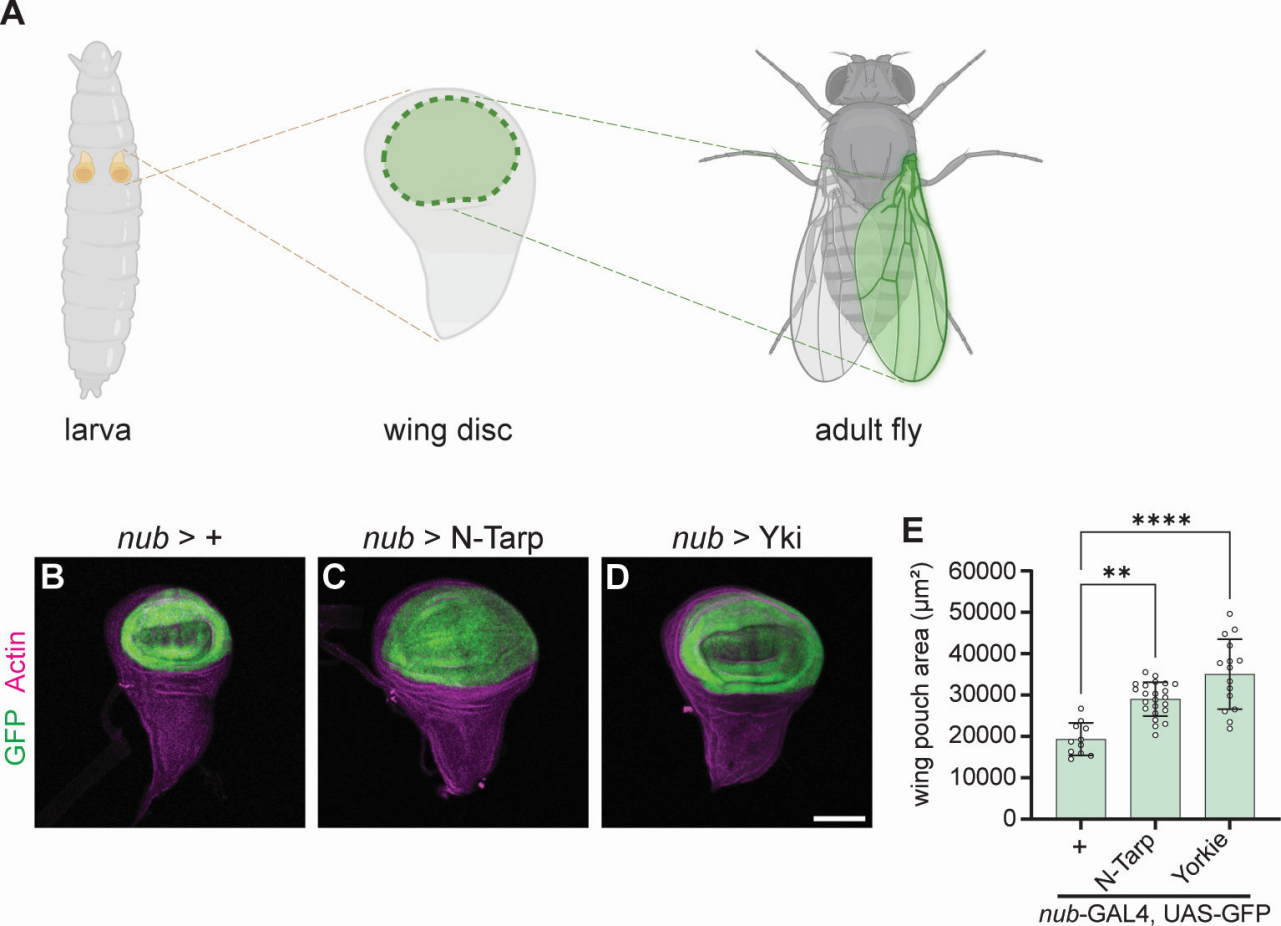
N-Tarp expression causes larval wing pouch overgrowth. (**A**) *Drosophila* larvae carry a pair of imaginal wing discs (left). The wing discs are larval tissue precursors of the adult dorsal thorax and wings. The pouch region of the wing disc (middle, green region) gives rise to the entire wing blade of the adult fly (right, green highlight). Created in BioRender. (**B–D**) Wing discs were dissected from third instar larvae and co-stained with phalloidin to label actin. *nub*-GAL4 drives expression of (**B**) GFP alone or together with (**C**) N-Tarp or (**D**) the Hippo pathway co-activator Yorkie (Yki). The scale bar represents 50 µm. (**E**) Wing pouch area measurement from control discs or discs expressing N-Tarp or Yorkie. Each hollow dot represents a wing disc. Bar graphs indicate the mean and SD. Kruskal-Wallis test with Dunn’s multiple comparisons was used. **P < 0.01, ****P < 0.0001.

Imaginal discs are larval tissue that serve as precursors to major structures in the adult fly, such as the antenna, eye, legs, and wings, among others. As the larva grows, the imaginal discs such as the wing disc also proportionally increase in size (25). This growth is driven by simultaneous cell proliferation and tissue patterning mediated via the Hippo pathway (26), making the wing imaginal disc an excellent tissue to study Hippo signaling. The pair of larval wing discs forms the complete dorsal thorax and wings. The wing pouch region of the larval wing disc (Fig. 1A, middle) gives rise to the adult wing blade (Fig. 1A, right).

We used *nubbin*-GAL4 (*nub*-GAL4) to express N-Tarp in the wing pouch region (Fig. 1B, GFP-expressing region). By measuring the area of GFP-positive tissue, we show that N-Tarp expression results in a larger wing pouch area compared to control (Fig. 1C and E). Overexpression of Yorkie, the Hippo pathway transcription co-activator, similarly results in increased wing pouch area (Fig. 1D and E), consistent with published findings (27, 28).

**Fig 2:**
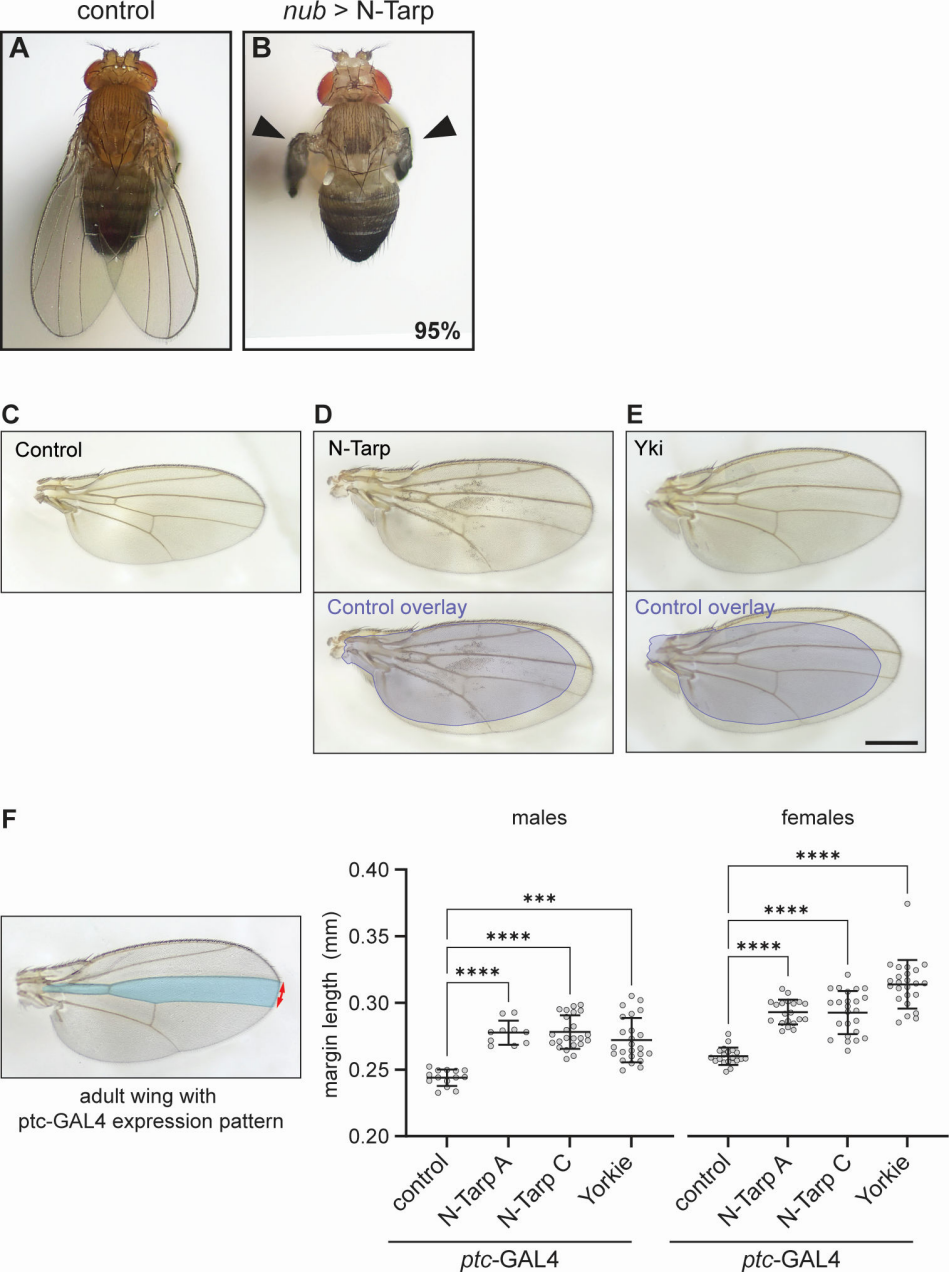
N-Tarp expression leads to increased wing size. (**A and B**) Dorsal view of an adult (**A**) control (*nub*-GAL4/+) fly or (**B**) *nub*-GAL4/UAS-N-Tarp fly. Black arrowheads indicate crumpled wings that occur in 95% of examined flies (*n* = 350 flies over three trials). (**C–E**) Dissected wing blades were mounted on slides and imaged. (**C**) Control wings are flat with stereotypical vein and crossvein patterns. (**D**) Five percent of *nub*-GAL4/UAS-N-Tarp flies have fully expanded wings (top panel). The bottom panel is the same *nub*-GAL4/UAS-N-Tarp wing with an overlay of the control wing outline (blue), revealing the difference in wing size. (**E**) Wing from *nub*-GAL4/UAS-Yorkie (Yki) flies are also larger compared to control. Scale bar is 500 µm. (**F**) (Left) *ptc*-GAL4 expression pattern (blue region) is limited to a small compartment of the adult wing. The distance between the top and bottom boundaries of the expression pattern was measured along the wing margin (red arrows). (Right) Scatter plot of wing margin length along the *ptc*-GAL4 expression boundary in control, UAS-N-Tarp, and UAS-Yorkie wings, indicating mean and SD. Two unique transgenic lines of UAS-N-Tarp (A and C) were tested, showing a similar increase in wing margin length. Yorkie expression also results in increased wing margin length. Wings from male and female adult flies were analyzed separately. The Kruskal-Wallis test with Dunn’s multiple comparisons was used. ***P < 0.001, ****P < 0.0001.

We then allowed *nub*>N-Tarp larvae to develop into adult flies to look at the impact on the adult wing blade. The wings of control adults are flat when fully expanded (Fig. 2A). Surprisingly, the vast majority (94.9%, *n* = 350 flies over three trials) of *nub*>N-Tarp adult flies have crumpled, unexpanded wings (Fig. 2B). The crumpled wings remain throughout the life of the adult flies. Counterintuitively, wing overgrowth has been observed to present as crumpled wings (28, 29). Indeed, the small proportion (5.1%) of *nub*>N-Tarp adult flies that have fully expanded wings displayed a much larger wing area (Fig. 2D) compared to control flies (Fig. 2C), consistent with the wing pouch overgrowth seen in *nub*>N-Tarp larval wing discs (Fig. 1). As expected, overexpression of Yki in the wing pouch also causes increased wing size (Fig. 2E). Interestingly, the crumpled wing phenotype was specific to N-Tarp expression and was not observed upon expression of full-length Tarp or another *C. trachomatis* early effector, TmeA (Fig. S1).

To increase the number of analyzable, fully expanded wings, we limited the expression of N-Tarp to a small compartment of the adult wing along the anterior/posterior boundary using the *ptc*-GAL4 driver (Fig. 2F, blue region in left panel) (30). Indeed, N-Tarp expression in a limited region of the wing did not interfere with wing expansion and resulted in expanded wings. We then measured the wing margin length between the anterior and posterior boundaries of the *ptc*-GAL4 domain (Fig. 2F, red arrow in left panel). Multiple, unique N-Tarp transgenic lines both resulted in increased margin length compared to control wings (Fig. 2F, graph). Moreover, this N-Tarp-induced increase in margin length phenocopies the increase caused by Yorkie overexpression (Fig. 2F), consistent with the observations from the larval wing disc.

A larger tissue size can arise from an increase in the total cell number from overproliferation, consistent with the role of the Hippo pathway. It can also occur from an increase in the individual cell size with no appreciable increase in cell number, as in the case of TOR signaling (31). To distinguish between the two possibilities, we quantified the cell density within the *ptc*-GAL4 compartment upon expression of N-Tarp or Yorkie overexpression by counting the number of trichomes, microscopic bristles that cover the entire wing surface, within a defined region (32). Trichomes originate from individual cells and act as a surrogate visual indicator of cell number. We observed no change in trichome density between control, N-Tarp-expressing, and Yorkie-overexpressing wings (Fig. S2), indicating that larger wing dimensions upon N-Tarp expression are likely due to increased cell number as a result of overproliferation.

### N-Tarp causes upregulation of Hippo pathway target genes

**Fig 3:**
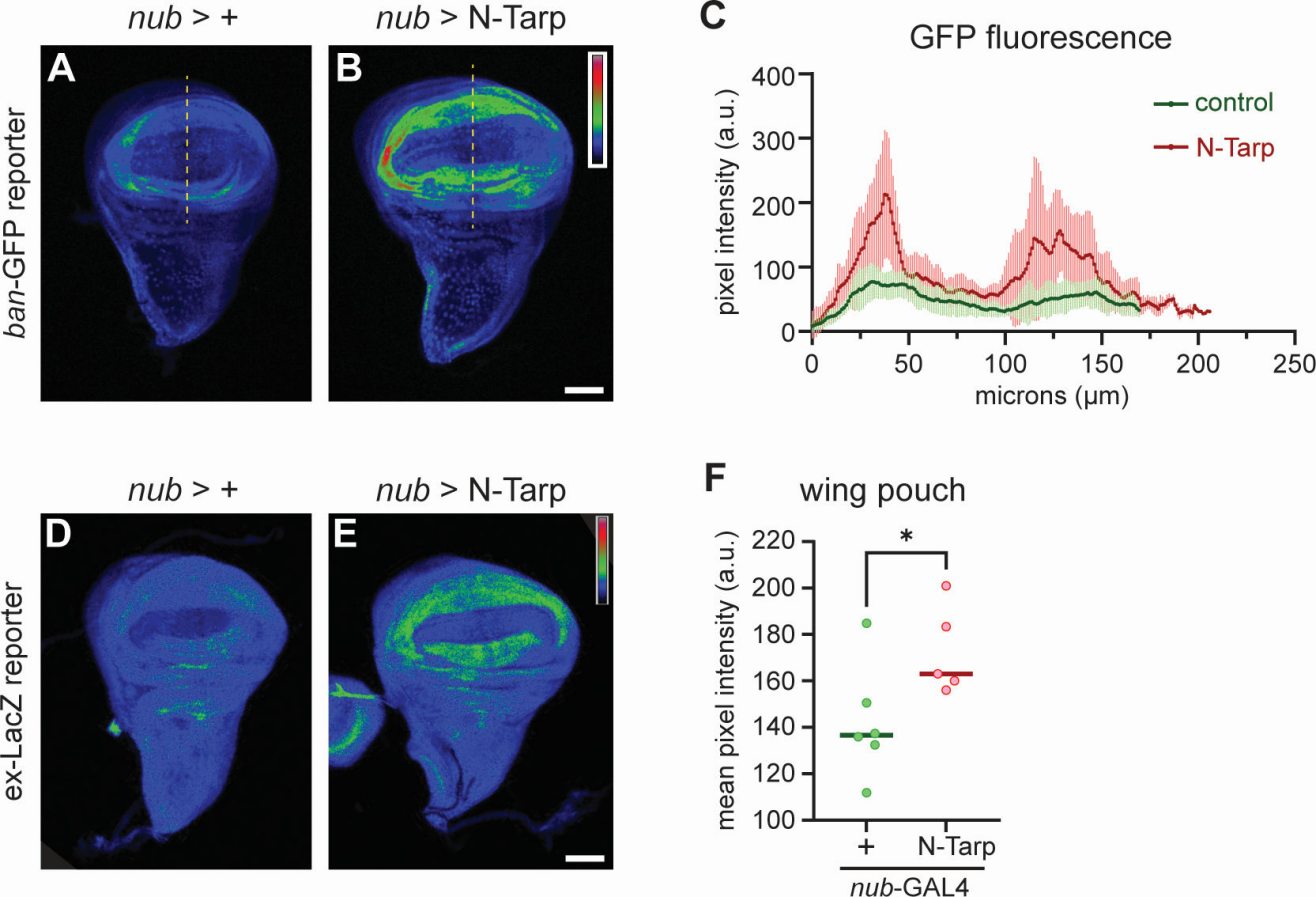
Hippo pathway target genes are upregulated upon N-Tarp expression. (**A–C**) The promoter of the Hippo pathway target gene *bantam* (*ban*) controls the expression of GFP (*ban*-GFP) and is used as a reporter of Hippo pathway activity in the wing discs. GFP fluorescence intensity is represented using a rainbow LUT. (**A**) Control (*nub*>+) wing discs display the baseline GFP intensity, while (**B**) N-Tarp expression in the wing pouch (*nub*>N-Tarp) results in increased GFP intensity. (**C**) Mean pixel intensity ± SD along a line ROI (yellow dashed lines) that traverses the wing pouch was plotted for control (*nub*>+, green; *n* = 22) and N-Tarp (*nub*>N-Tarp, red; *n* = 17). (**D–F**) Another Hippo pathway reporter uses the promoter of the Hippo target gene expanded (*ex*) to drive LacZ expression (*ex*-LacZ). LacZ levels were visualized by immunostaining and displayed using a rainbow LUT. (**D**) Control (*nub*>+) wing discs show baseline LacZ levels, while (**E**) N-Tarp expression in the wing pouch (*nub*>N-Tarp) results in elevated LacZ levels. (**F**) The mean pixel intensity ± SD within the wing pouch region for control (*nub*>+, green) and N-Tarp (*nub*>N-Tarp, red) is plotted. Welch’s test used. *P < 0.05. Scale bar is 50 µm.

Above, we show that N-Tarp-induced tissue overgrowth in the larval wing disc and in the adult wing phenocopies changes brought on by Yorkie overexpression. To further support the link between N-Tarp and the Hippo pathway, we sought to determine whether the expression of canonical Hippo target genes is also altered in the presence of N-Tarp. The expression of the gene *bantam* is controlled by the Hippo pathway (33, 34). We used a *bantam*:GFP reporter (35) to query Hippo pathway activity in developing wing discs upon expression of N-Tarp. Control wing discs display an overall low GFP fluorescence in the wing pouch, with only very small regions of moderate activity (Fig. 3A). On the other hand, N-Tarp expression in the wing pouch resulted in a striking increase in *bantam* gene expression, as evidenced by the expanded area of moderate GFP fluorescence intensity and the appearance of high-activity regions (Fig. 3B). Quantitative analysis of GFP fluorescence intensity along a linear region of interest (ROI) (Fig. 3A and B, dashed line) shows a consistently increased level of *bantam* gene expression upon N-Tarp expression over multiple wing discs examined (Fig. 3C). Instead of measuring GFP intensity over the wing pouch region, a linear ROI was chosen to remain independent of the different wing pouch sizes between the two genotypes (Fig. 1E). A transgenic reporter of another canonical Hippo pathway target gene, *expanded* (36), also showed similar results (Fig. 3D through F). In all, the above results provide molecular proof that N-Tarp expression alters Hippo pathway activity.

### N-Tarp-induced wing overgrowth is rescued by disrupting the Hippo pathway

To prove that N-Tarp function acts through the Hippo pathway, we attempted to rescue the crumpled wing phenotype (Fig. 2B) by either disrupting (i) the Yki transcription factor or (ii) the canonical Hippo target genes *Diap1* and *CycE*. Together, *Diap1* and *CycE* can promote tissue growth through overproliferation. If N-Tarp’s *in vivo* activity is indeed acting through the Hippo pathway, then disrupting either the key transcription factor, Yki, or the canonical Hippo target genes linked to cell proliferation should result in amelioration of the crumpled wing phenotype.

**Fig 4:**
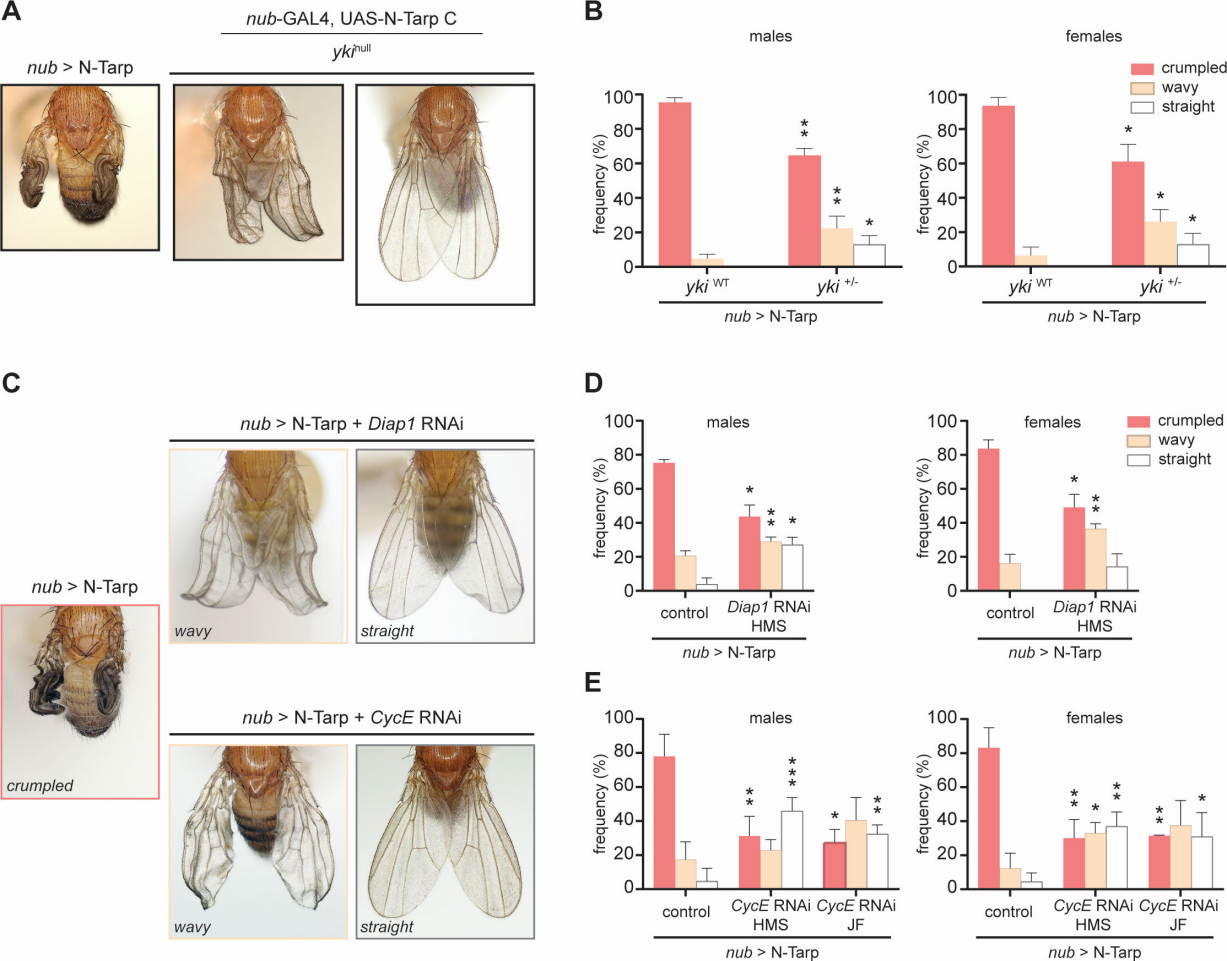
N-Tarp-induced overgrowth rescued by disrupting the Hippo pathway. N-Tarp was expressed in the developing wing on its own (*nub*>N-Tarp) or in the genetic background of (**A and B**) heterozygote yki^null^ mutant or (**C–E**) RNAi knockdown of the Hippo target genes *Diap1* or *CycE*. For each condition, the severity of wing phenotypes was categorized and scored as crumpled (strong), wavy (intermediate), or straight (normal). Representative images of wing phenotypes are displayed (**A and C**). Expression of N-Tarp in the developing wing results in a crumpled wing phenotype in 77%–95% of the progeny (B, yki^WT^; D and E, control). When expressed in a heterozygous yki^null^ background, there is a significant decrease in the proportion of flies displaying crumpled wings and a concomitant increase in the flies displaying an intermediate wavy wing phenotype and the appearance of flies with normal wings (B, yki^+/-^). Expression of N-Tarp in a *Diap1* RNAi or *CycE* RNAi background similarly caused a drop in crumpled wing frequency and a shift toward the intermediate wavy and normal wing category (**D and E**). HMS and JF refer to different *Drosophila* RNAi collections. Statistical tests were performed per phenotypic category. More details are included in Materials and Methods. *P < 0.05, **P < 0.01, ***P < 0.001. Four independent trials of the genetic rescue experiments were performed.

We expressed N-Tarp in the wing pouch of *yki*^+/-^ heterozygous null flies. Reduced *yki* gene dosage leads to reduced Yki protein levels (37) without dramatically affecting animal viability; the use of RNAi to knock down expression of *yki* in the wing pouch leads to strong lethality (data not shown). We then measured the frequency of observing N-Tarp-induced crumpled wings in the wild-type background vs the *yki*^+/-^ background. As expected, expression of N-Tarp in the wild-type background results in all flies with abnormal wings with a high frequency of crumpled wings (Fig. 4A and B [left panel]). Expression of N-Tarp in the *yki*^+/-^ background results in reduced crumpled wing frequency, an increase in an intermediate, wavy wing phenotype frequency, and, more importantly, the appearance of normal, straight wings (Fig. 4A (middle and right panels) and B). We also expressed N-Tarp in the wing pouch while simultaneously knocking down *Diap1* or *CycE* expression by RNAi. Knockdown of either *Diap1* or *CycE* shifts the distribution of N-Tarp-induced wing phenotypes more strongly toward the intermediate wavy wing or fully straight wings (Fig. 4C through E). Flies that are solely heterozygous null for Yki or underwent RNAi knockdown of *Diap1* or *CycE* in the wing pouch all have straight wings (Fig. S3).

A shift from predominantly crumpled wings to partially or fully expanded wings upon disruption of the Hippo pathway, as performed above, strongly indicates that N-Tarp exerts its function *in vivo* by acting through the Hippo pathway. The essential nature of Hippo pathway activity to animal viability precludes the examination of N-Tarp function in a completely null Yki, *Diap1*, or *CycE* background, which can explain the partial nature of the genetic rescue.

**Fig 5:**
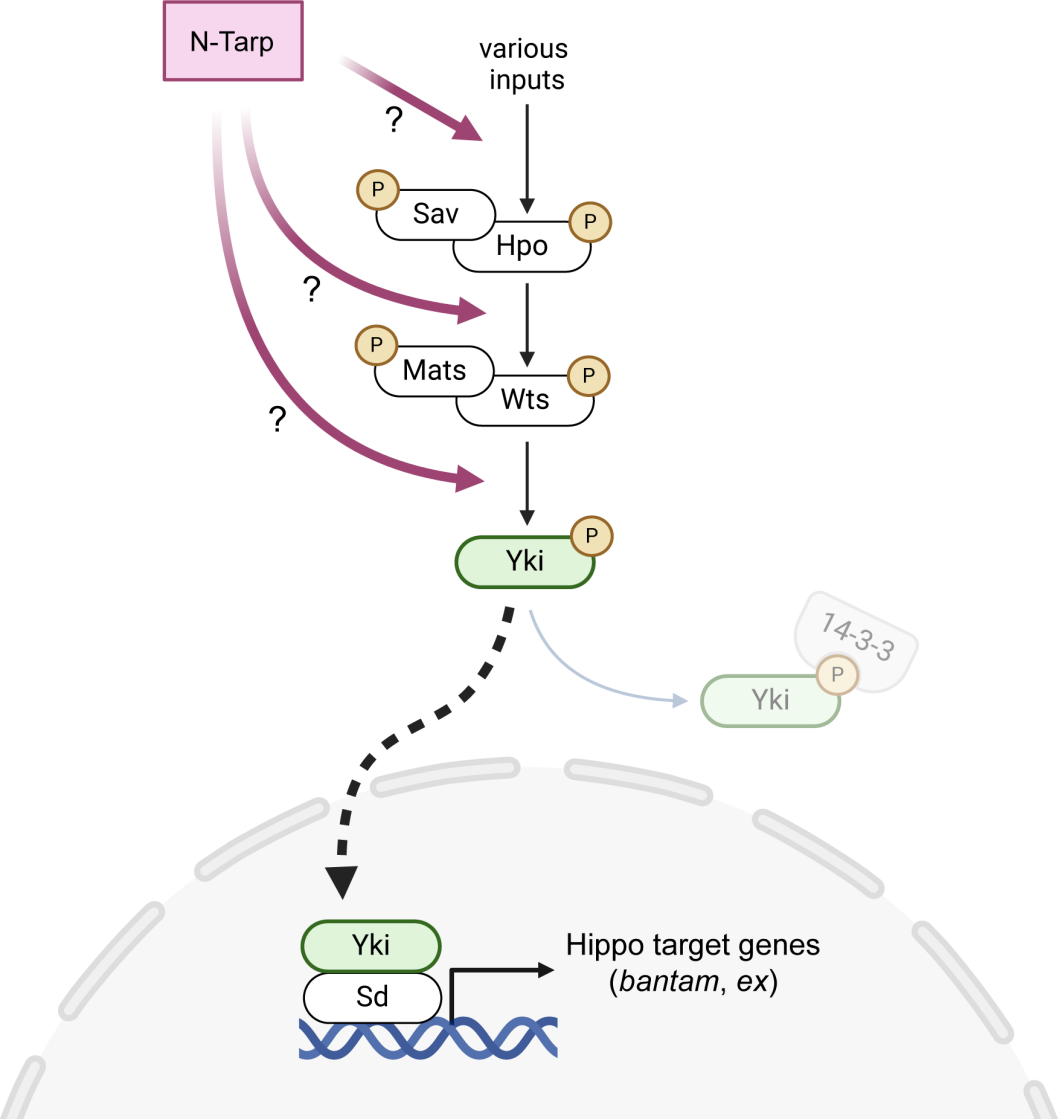
The N-terminal region of the *Chlamydia* effector Tarp acts on the host Hippo pathway to increase target gene expression. The core kinase cascade of the host Hippo pathway is made up of the following proteins: Salvador (Sav), Hippo (Hpo), Mob as tumor suppressor (Mats), and Warts (Wts). The Hippo pathway incorporates multiple signaling inputs to set the phosphorylation state of the transcriptional co-activator Yorkie (Yki). Phosphorylated Yki binds to the 14-3-3 protein and is retained in the cytoplasm. Unphosphorylated Yki translocates into the nucleus and binds to the TEAD protein Scalloped (Sd), which then acts as a transcription factor to turn on Hippo target gene expression. N-Tarp acts upstream of the Yki co-activator to cause increased expression of canonical Hippo target genes (*bantam* and *ex*) but whether it acts directly on the core signaling components or as an upstream input is not yet determined. Created in BioRender.

In summary, using *Drosophila* cell and developmental biology as a platform, we present strong evidence that the N-terminal region of Tarp alters the activity of the host Hippo signaling pathway upstream of the co-activator Yorkie, resulting in the increased expression of Hippo target genes (Fig. 5). This agrees with our previous study that shows a Tarp-dependent change in Hippo pathway activity in an *in vitro* infection model (19). Finally, this work provides ample evidence of a novel function of the N-terminal region of Tarp and positions Tarp as having an important role outside of host cell invasion.

## DISCUSSION

*Drosophila melanogaster* is an established model organism with a rich repertoire of genetic tools, reagents, and techniques. Perturbations caused by bacterial effector expression in functional living tissue, in contrast to cells *in vitro*, often result in abnormal development of organs or the whole organism itself and are readily determined by visual inspection. Because the cell and developmental biology of *Drosophila* is well documented, researchers can link a developmental phenotype to the relevant gene or signaling pathway. Thus, *Drosophila i*s an effective discovery tool for bacterial effector function, and its versatility facilitates continued functional studies.

Though there is a high degree of conserved genes and pathways between *Drosophila* and mammals, studying bacterial effectors that specifically target mammalian proteins that are divergent from or absent in flies will not be fruitful. Also, some effectors may only function in the correct context (subcellular localization and interaction with other effectors) and, without prior knowledge, might not be recapitulated during transgenic expression. Lastly, some effector functions might not disrupt cell function at all and cause no appreciable phenotypes. Despite these caveats, cloning *Chlamydia* effector genes, generating transgenic *Drosophila*, and initial phenotypic characterization are routine and less labor and resource intensive than generating and characterizing new *Chlamydia* mutants. Thus, using *Drosophila* as a platform is a positive addition to the toolkit that researchers use to attribute function to the long list of uncharacterized *Chlamydia trachomatis* effectors.

This work takes advantage of the genetic tools and techniques available to *Drosophila* researchers to firmly establish the link between N-Tarp and the Hippo pathway using morphological, molecular, and genetic approaches. In particular, the demonstration of genetic rescue of the N-Tarp-induced wing phenotype conclusively shows that N-Tarp acts through the Hippo pathway, placing it upstream of the co-activator Yorkie. Regulation of the Hippo pathway is complex, with multiple upstream inputs as well as direct influence on intermediate components (38). As such, further efforts to determine the molecular mechanism of N-Tarp activity will involve identifying the exact point of intersection with the Hippo pathway (Fig. 5).

The function of the N-terminal region of Tarp is not well understood. The early effector Tarp is secreted into the host cell to promote host cell invasion after *Chlamydia* attachment to the cell surface (6, 8). The importance of Tarp in the invasion process is highlighted by its evolutionary conservation across multiple *Chlamydia* spp. that target a wide spectrum of host animals, ranging from mammals, birds, and reptiles to fish (15). The C-terminal region of Tarp harbors actin-interacting domains that are important for actin nucleation, polymerization, and filament bundling (4, 10, 11), whereas the N-terminal region is mostly devoid of any annotated domains. The exception to this is the human pathogen *C. trachomatis*, whose Tarp N-terminus can contain varying numbers of tyrosine-rich repeats, depending on the serovar (17).

The presence of a Tarp N-terminal region is observed across multiple *Chlamydia* spp. (15), a telltale sign of a significant but underappreciated role in infection. *C. trachomatis* Tarp is rapidly phosphorylated at the N-terminus upon delivery into the host cell during the attachment and early invasion steps of infection (8, 39). Similarly, when expressed in *Drosophila* tissue, we observe phosphorylation of full-length Tarp (18), and the N-Tarp fragment is phosphorylated when incubated in *Drosophila* lysate (Fig. S4). Phosphorylation is a powerful signaling cue, and peptides that represent N-Tarp phosphorylation have been shown to bind strongly to human SHC1, an SH2 domain-containing protein, to activate MEK/ERK signaling and drive downstream gene expression related to cell survival (40).

*Chlamydia*-infected cells in culture are surprisingly resistant to extrinsic and intrinsic triggers of apoptosis (41). *Chlamydia* development takes 24–48 hours to complete, depending on the host cell type. This is ample time for the host cell to mount a defense response and, failing that, to undergo apoptosis. Once triggered, apoptosis progresses rapidly, resulting in cell death in a shorter time frame. However, apoptosis in *Ct*-infected cells is blocked at multiple levels—there is no activation of Bcl-2 family members Bax and Bak (42, 43), no release of cytochrome c from mitochondria, and a lack of processing of caspases 9 and 3 (44)—though it is not clear how this is accomplished. The work by Mehlitz et al. (40) as well as studies focused on another *C. trachomatis* effector, CpoS, both address the search for *Chlamydia*’s anti-apoptotic effectors (45).

Inhibitor of apoptosis (IAP) proteins are negative regulators of apoptosis and cell death that can block apoptosis (46). Interestingly, the human and *Drosophila* IAPs *XIAP* and *Diap1*, respectively, are canonical target genes of the host Hippo pathway. It was recently shown that Hippo pathway activity is altered during *Ct* infection *in vitro*, resulting in increased nuclear localization of the co-activator YAP (47), as well as increased *XIAP* gene expression (19). Moreover, the increase in *XIAP* expression is dependent on the presence of Tarp (19). Our study extends that discovery by implicating the N-terminal region of *Ct* Tarp as sufficient to alter Hippo signaling using an *in vivo* model. Taken together, it is possible that *Chlamydia*, via the N-terminal region of Tarp, also engages the Hippo pathway to increase IAP levels to ensure host cell survival during infection.

The Hippo pathway crosstalks with other host signaling pathways, apart from its classic role in controlling host cell survival. Therefore, it is not surprising that many other pathogens have evolved strategies to directly manipulate the host Hippo pathway to promote infection. *Legionella pneumophila* secretes the effector LegK7, which directly phosphorylates MOB1 (Mats in flies), altering the host transcriptional response to promote infection (48). MOB1/Mats is a member of the core kinase cascade of the Hippo pathway and is normally phosphorylated by the upstream Hippo kinase. *Ehrlichia chaffeensis* secretes TRP120, which turns down Hippo signaling, leading to expression of the anti-apoptotic Hippo target gene *SLC2A1*/*GLUT1* (49). Viral infections have also targeted Hippo signaling. The human papillomavirus (HPV) E6 protein directly interacts with multiple members of the Hippo pathway (50, 51), contributing to the formation of the poorly differentiated tissue lesions associated with HPV infections. *Helicobacter pylori*, as well as many other viruses, have documented changes in Hippo signaling, although the mediating effector or viral protein might act indirectly or has not been elucidated at this time (52, 53).

Hippo pathway perturbation is an important contributor to carcinogenesis, including gynecological malignancies (54). Genomic analysis of clinical samples has been used to look at the state of Hippo pathway signaling in the context of cancer (54). This includes measuring gene amplification, mutation, or deletions at the genome level as well as gene expression at the mRNA level. Similar molecular diagnostic approaches can be used on clinical samples from *Chlamydia*-infected patients to determine infection-induced changes in the Hippo pathway.

This work supports a role for Tarp, via its N-terminal region, as the mediating effector that intersects with the host Hippo pathway. The engagement of Hippo signaling during *C. trachomatis* infection is a newly emerging avenue of inquiry that may hold answers to long-standing questions of resistance to apoptosis as well as pathological changes in infected tissue *in vivo*.

## MATERIALS AND METHODS

### *Drosophila* stocks, handling, and rearing

From the genomic DNA of *Ct* serovar L2 strain 434/Bu (ATCC VR-902B), the open reading frame that encodes for the N-terminal region of *Ct* L2 Tarp (N-Tarp, amino acids 1–431) was cloned into the pUAST *Drosophila* transformation and expression vector and used for P-element-mediated germline transformation (model system injections) to generate UAS-N-Tarp transgenic flies (55).

Transgene expression was performed using the modular GAL4/UAS binary expression system (55). Briefly, the yeast-derived GAL4 transcription factor binds to its cognate UAS promoter sequence, driving transgene expression. In practice, UAS-transgene flies are crossed with GAL4 flies to generate progeny that express the transgene of interest in the target tissues.

We previously generated and validated the transgenic expression of N-Tarp (19), as well as full-length Tarp and the early effector TmeA (18) in flies.

Other stocks used were derived from or directly obtained from the Bloomington *Drosophila* Stock Center (BL) or were kind gifts from other scientists: *ban*-GFP; *nub*-GAL4 (*ban*-GFP is a gift from I. Hariharan), *ex*-lacZ/CyO; TM2/TM6B (BL-44248), *nub*-GAL4, UAS-GFP (*nub*-GAL4 derived from BL-86108), *nub*-GAL4, UAS-N-Tarp; TM6 tubGAL80/+, *ptc*-GAL4 (BL-2017), UAS-yki:GFP (BL-28815), UAS *Diap1* RNAi HMS00752 (BL-33597), UAS *CycE* RNAi HMS00060 (BL-33654), UAS *CycE* RNAi JF02473 (BL-29314), w1118 (BL-5905), yki[B5]/CyO (BL-36290).

The cross to generate *ptc*-GAL4/UAS N-Tarp flies for compartmentalized N-Tarp expression in the adult wing was reared at 18°C to improve progeny survival. All fly stocks and other crosses were reared at 25°C. Flies were grown on Nutri-fly BF media (Genesee Scientific) supplemented with 0.45% vol/vol propionic acid as mold inhibitor. We used FlyBase (release FB2024_02) to obtain information on phenotypes, expression patterns, and available stocks (56).

### Immunostaining and imaging of imaginal wing discs

Wing imaginal discs were dissected from third instar larvae in phosphate-buffered saline (PBS) and transferred into a watch glass well. Tissue fixation was performed using 4% formaldehyde in PBS-Triton X-100 (0.1% vol/vol) (PBT) for 10 min while rocking on a small orbital shaker. Fixed tissue was blocked in PBT-bovine serum albumin for 1 hour prior to immunostaining. The primary antibody used was 1:50 anti-LacZ (40–1a, DSHB). The secondary antibody used was 1:400 anti-mouse Alexa Fluor 594 (Invitrogen). The co-stains used were 1:400 phalloidin Alexa Fluor 568 (Invitrogen), 1:400 GFP-Booster (Chromotek), and 4′,6-diamidino-2-phenylindole (Invitrogen). Stained wing discs were mounted onto glass slides using Aqua-polymount and placed under a cover glass (#1.5).

A Zeiss LSM 710 confocal microscope was used to acquire fluorescent images of immunostained wing discs using a 10× EC Plan-Neofluar objective. Imaging of *ban*-GFP and *ex*-LacZ reporter intensities was performed with identical acquisition settings between control and experimental samples.

### Measuring wing pouch size

GFP expression driven by *nub*-GAL4 visually delineates the wing pouch region. Z-stacks of confocal images of wing discs were z-projected using the “maximum intensity” setting. The region of interest defined by GFP expression was selected, and the ROI area was measured. Image analysis was performed using FIJI (57).

### Scoring and imaging of wing phenotypes in adult flies

Adult fly wings were visually examined under CO_2_ anesthesia using a Leica stereomicroscope and categorized and counted as having crumpled, wavy, or straight wings (representative images in Fig. 4). Statistical tests were performed per phenotypic category between genotypes. Comparisons between two genotypes (as in Fig. 4B and D) were performed using paired *t*-tests, while comparison across three genotypes (as in Fig. 4E) was performed using Brown-Forsythe and Welch analysis of variance tests with Dunnett’s T3 multiple comparisons.

Flies were mounted on wooden picks for imaging attached wings. To analyze wing dimensions, wings were clipped at the hinge, fixed in 70% ethanol, and mounted on glass slides with a small amount of 40% glycerol. Whole flies or clipped wings were imaged using either a Zeiss Stemi 508 stereomicroscope with an Axiocam 208 color camera or a Keyence VHX-7000 digital microscope. Measurement of wing dimensions was performed using FIJI (57).

For quantifying trichome number in the wing, a defined square ROI was overlayed within the *ptc*-GAL4 wing compartment, close to the wing margin (see Fig. S3), and the number of trichomes within the ROI was counted visually.

Where applicable, male and female adult flies were assessed separately in accordance with National Institutes of Health recommended practice to consider organism sex as a biological variable.

### Measuring Hippo pathway activity using genetically encoded reporters

To measure the Hippo pathway activity, we utilized two genetically encoded Hippo pathway reporters: *ban*-GFP (35) and *ex*-LacZ (58). N-Tarp was expressed in the wing disc using *nub*-GAL4 in the genetic background of either *ban*-GFP or *ex*-LacZ. The wing disc were dissected and immunostained for either GFP (using GFP-Booster) or LacZ and imaged using confocal microscopy (see Materials and Methods above). Z-stacks were z-projected using the “sum slices” setting to generate a cumulative image of reporter intensity through the entire wing disc thickness. For *ban*-GFP, the pixel intensity was recorded along a line ROI placed on the wing disc midline. For *ex*-LacZ, the mean pixel intensity within an area ROI delineating the wing pouch region was measured. Image analysis was performed using FIJI (57).

### *In vitro* kinase assay of purified N-Tarp

GST-tagged N-Tarp (GST-Tarp^1–625^) was expressed in *Escherichia coli* and purified using glutathione affinity purification as previously described (11). *Drosophila* lysate was obtained by mechanical disruption of frozen flies with mini mortars and pestles followed by sonication in 100 mM KCl, 2 mM MgCl2, 1 mM ATP, and 10 mM HEPES (pH 7.4) (Buffer A). Insoluble *Drosophila* material was cleared by centrifugation (20,000 × *g*, 20 min, 4°C). Purified N-Tarp and a GST control immobilized on glutathione beads were incubated with the *Drosophila* lysate for 1 hour at 20°C. Following the incubation, the beads were washed 3× with buffer A and resuspended directly into a Laemmli sample buffer. Samples were analyzed by SDS-PAGE followed by Coomassie stain or Western blot for tyrosine phosphorylation (4G10 mouse monoclonal antibody, EMD Millipore Corp.).

### Graphs, figures, and statistics

Graphs and statistical analyses were generated and performed using GraphPad Prism version 10. Representative images and schematics were prepared using FIJI, Adobe Photoshop, and BioRender. Figures were assembled in Adobe Illustrator.

## SUPPLEMENTAL MATERIAL

Supplemental material spectrum.02596-24-s0001.docx Fig. S1 to S4.

ASM does not own the copyrights to Supplemental Material that may be linked to, or accessed through, an article. The authors have granted ASM a non-exclusive, world-wide license to publish the Supplemental Material files. Please contact the corresponding author directly for reuse.
