# Supplementary material for: The N-terminus of the Chlamydia trachomatis effector Tarp engages the host Hippo pathway
Source: Microbiol Spectr. 2025 Mar 10;13(4):e02596-24. doi: 10.1128/spectrum.02596-24 (PMC11960468; doi:10.1128/spectrum.02596-24)
Supplement: Supplemental material — Fig. S1 to S4. [file spectrum.02596-24-s0001.docx]

**Supporting Information**

**
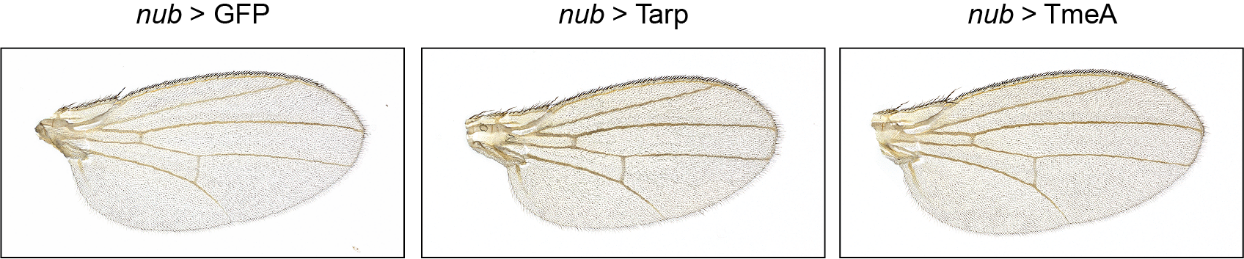
**

**Figure S1. Expression of *Chlamydia* effectors Tarp (full-length) or TmeA does not disrupt wing morphology.** Representative wing images from flies expressing green fluorescent protein (GFP), full-length Tarp, and another *C. trachomatis* early effector, TmeA, in the developing wing pouch using *nub*-GAL4.


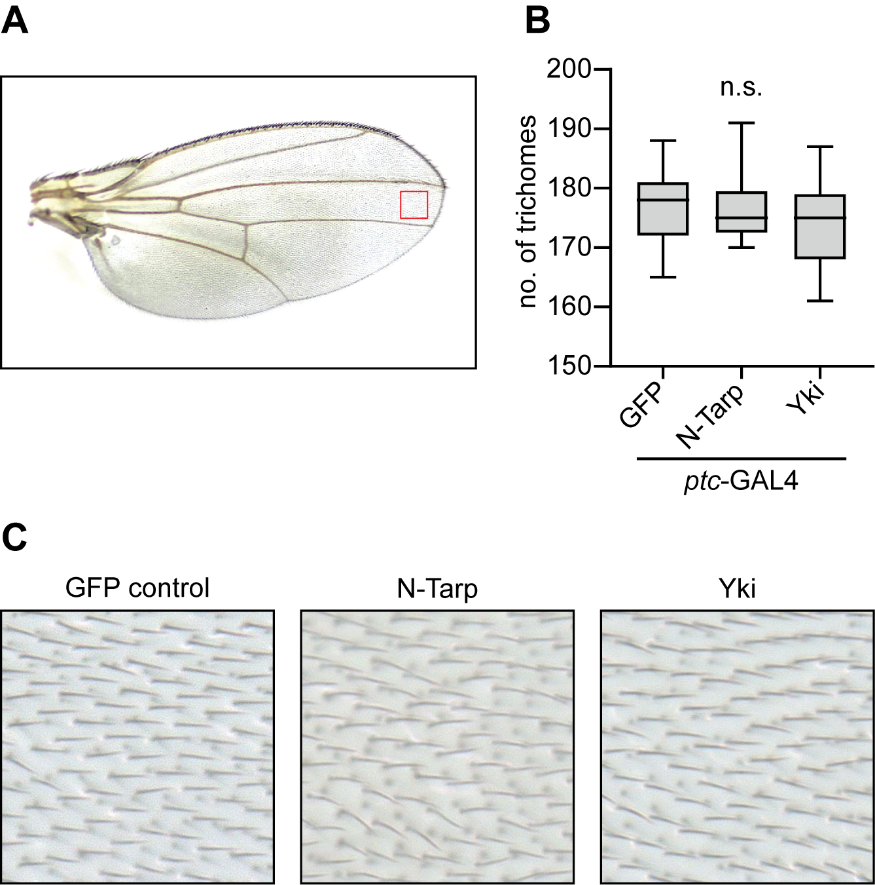


**Figure S2. Trichome density in the wing is not affected by the expression of
N-Tarp or Yorkie.** (A) A representative control wing displaying the region analyzed for trichome density (red box). (B) Box and whisker plots represent the number of trichomes (microscopic wing bristles) within the defined analysis region (red box) in wings expressing GFP (negative control), N-Tarp, or Yorkie (Yki) driven by *ptc*-GAL4. There is no statistical difference (n.s.) between the groups (Kruskal-Wallis test, n≥10 per group). (C) Representative, high magnification images of trichomes within the defined analysis region from wings expressing GFP (negative control), N-Tarp, or Yorkie (Yki) driven by *ptc*-GAL4.

**
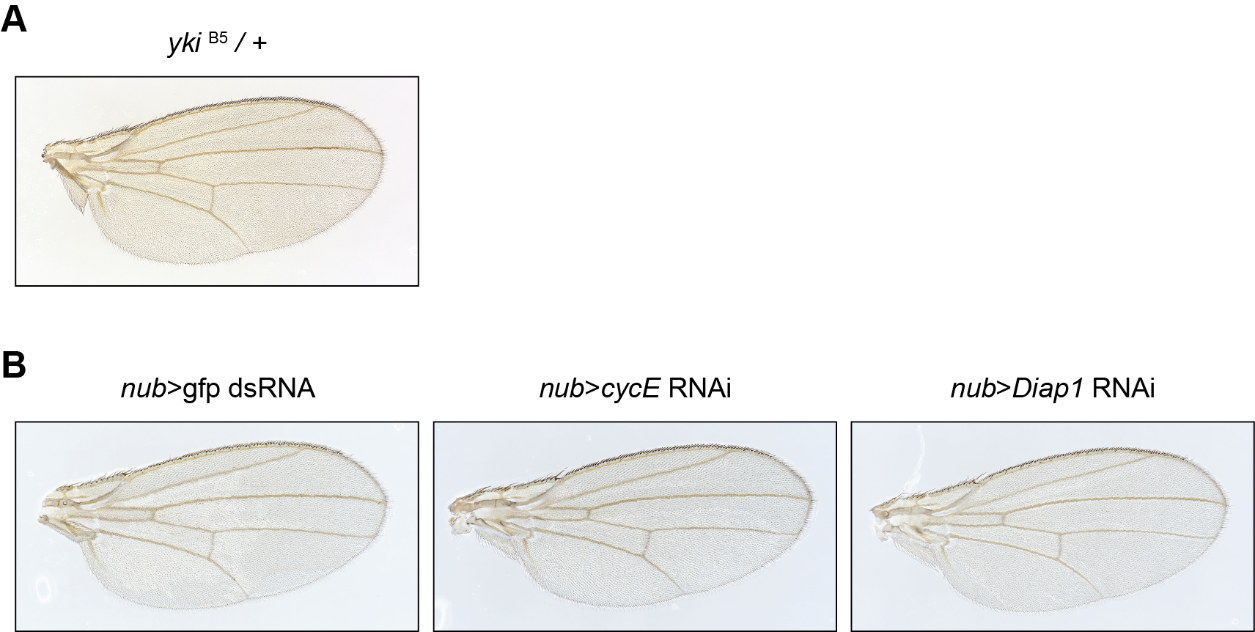
**

**Figure S3. Altering the levels of Yorkie, Diap1, or CycE alone does not disrupt wing morphology.** (A) Normal appearance of a wing from a heterozygote Yorkie null (yki^B5^/+) fly. (B) A non-targeting RNAi control (gfp dsRNA) does not alter the wing morphology. RNAi knockdown of *CycE* or *Diap1* in the wing disc pouch using *nub*-GAL4 also does not disrupt wing morphology.


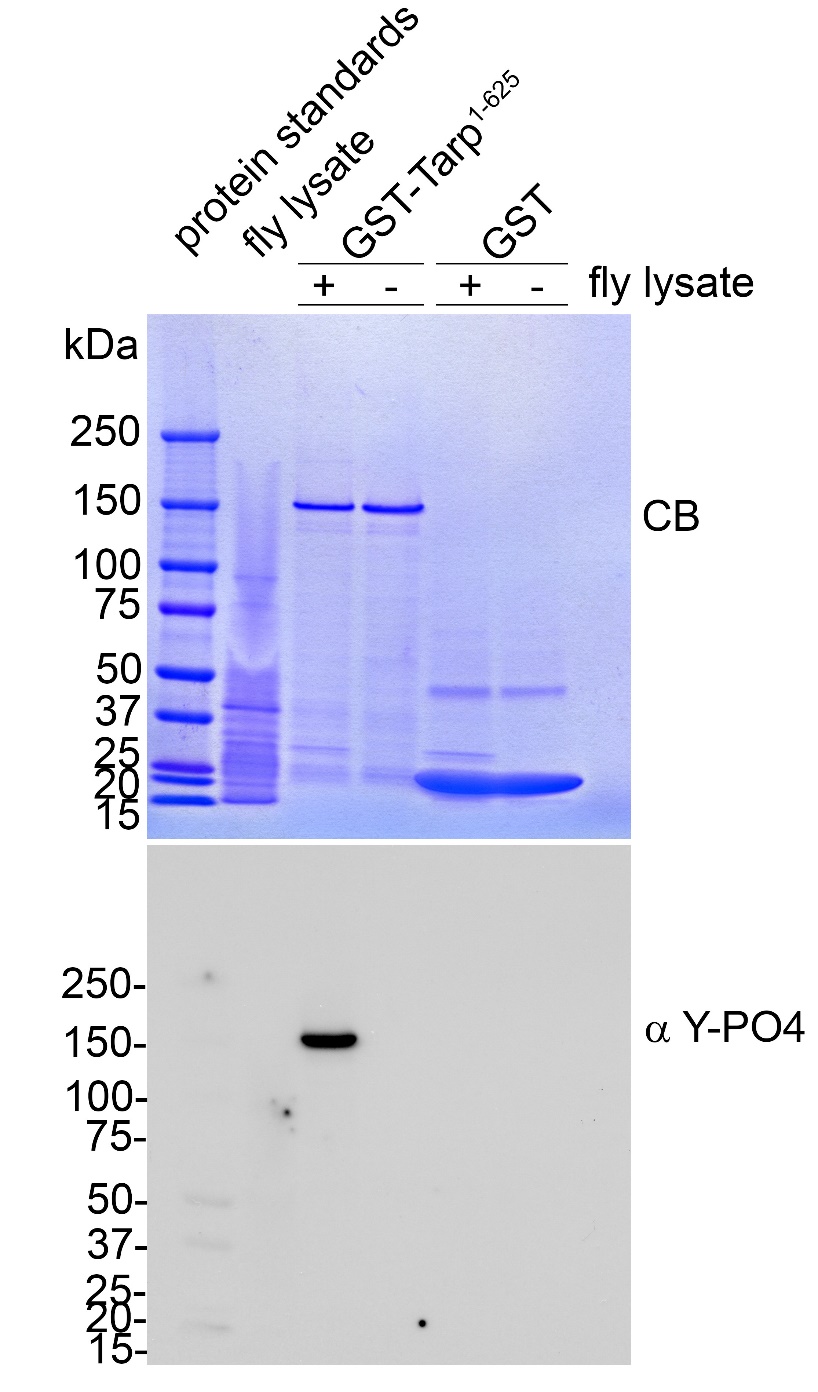


**Figure S4. Endogenous *Drosophila* kinases can phosphorylate N-Tarp.** (Top panel) Coomassie stain (CB) following SDS-PAGE validating the presence and relative amounts of the indicated samples. (Bottom panel) Western blot analysis of an identically loaded SDS-PAGE gel testing for the presence of tyrosine phosphorylation (α Y-PO4). Purified N-Tarp (GST-Tarp^1-625^) is tyrosine phosphorylated upon incubation with *Drosophila* lysate.
